# The correlation between Th17/Treg immune dysregulation and the disease severity in chronic spontaneous urticaria patients

**DOI:** 10.1002/iid3.920

**Published:** 2023-07-27

**Authors:** Xiaojing Yang, Leigang Chen, Shining Wang, Yuanhui Wu, Xiangzhao Zhou, Zhaoying Meng

**Affiliations:** ^1^ Department of Dermatology The First Affiliated Hospital of Hebei North University Zhangjiakou City Hebei Province China

**Keywords:** chronic spontaneous urticaria, disease severity, IL‐17, IL‐21, IL‐35, immune dysregulation, quality of life, TGF‐β1, Th17/Treg

## Abstract

**Objective:**

Chronic spontaneous urticaria (CSU) has a profound impact on the sleep quality, productivity and overall quality of life of affected individuals. This study aimed to investigate the correlation between serum Th17/Treg immune dysregulation and the severity of CSU in patients.

**Methods:**

Clinical baseline data of 120 CSU patients and matched healthy controls were recorded. The pruritus level, disease severity, and quality of life of CSU patients were assessed using the visual analogue scale, weekly Urticaria Activity Score and chronic urticaria quality of life questionnaire, respectively. The Th17/Treg cell ratio was detected by flow cytometry. ELISA was used to measure the levels of serum Th17 cytokines (IL‐17, IL‐21) and Treg cytokines (TGF‐β1, IL‐35). Pearson's correlation analysis was conducted to examine the associations between these indicators.

**Results:**

No significant differences were identified in terms of sex, age, and BMI between the two groups. However, CSU patients exhibited a significant increase in the Th17 cell ratio, as well as the elevated serum levels of TGF‐β1, IL‐17 and, IL‐21. Conversely, the proportion of Treg cells and the levels of IL‐35 were remarkably decreased in CSU patients. Peripheral blood Th17 cells were negatively correlated with Treg cells. The severity of pruritus, life quality, and disease severity in CSU patients were positively correlated to Th17 cell ratio, and inversely correlated with Treg cell proportion.

**Conclusions:**

A positive correlation was found between the percentage of peripheral blood Th17 cell in CSU patients and the pruritus level, life quality, and disease severity. In constrast, there was a negative correlation between the proportion of peripheral blood Treg cells and these clinical parameters.

## Graphical abstract

Chronic spontaneous urticaria (CSU) severely affects the quality of sleep, productivity and quality of life of patients. This study investigated the correlation between serum Th17/Treg immune dysregulation and the disease severity in CSU patients.

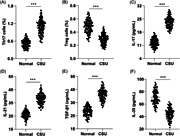

## 1 INTRODUCTION

Chronic urticaria (CU), a prevalent skin disorder characterized by the recurring occurrence of angioedema and wheals lasting for more than 6 weeks.^1^ CU can be classified into chronic spontaneous urticaria (CSU) and chronic inducible urticaria based on the cause and duration of the disease, with CSU being the most prevalent form accounting for approximately 30%–40% of all CU cases.^2^, ^3^ CSU is associated with a high morbidity, frequent relapses, and prolonged disease course, significantly impacting the quality of life of affected individuals. Despite its clinical significance, the exact underlying mechanisms responsible for CSU remains unclear.^4^ Therefore, further research is needed to elucidate the pathogenesis of CSU and identify the precise causative factors.

The immune theory is widely accepted among researchers as a possible explanation for the pathogenesis of CSU.^5^ According to this theory, CSU development is closely linked to immune dysregulation,^6^ characterized by abnormal activation of immune cells and excessive production of pro‐fibrotic and pro‐inflammatory molecules.^7^ Among the immune cells, CD4^+^ helper T (Th) cells play a significant role in CSU. These cells can differentiate into four subpopulations: Th1, Th2, Th17, and regulatory T (Treg) cells, with each subpopulation exhibiting a specific cytokine production and functions.^8^ While the Th1/Th2 cytokine imbalance in CSU pathogenesis has been extensively investigated,^9^, ^10^ the role of Th17/Treg immune dysregulation has received less attention. Treg cells and Th17 cells have antagonistic functions and work together to maintain immune homeostasis.^11^ CSU is characterized by autoimmune dysfunction with multifactorial involvement.^12^ Recent studies have revealed the importance of Th17/Treg imbalance in the progression of several autoimmune diseases and inflammatory diseases, such as systemic lupus erythematosus^13^ and autoimmune thyroid disease.^14^ Studies have identified an elevated Th17 expression in CSU, while Treg cells may contribute to the autoimmune process.^15^, ^16^ Therefore, Th17/Treg immune dysregulation likely plays a crucial role in CSU pathogenesis. However, the correlation between serum Th17/Treg immune dysregulation of CSU patients and the severity of CSU has not been investigated. Thus, this study aims to explore the correlation between serum Th17/Treg immune dysregulation and disease severity in CSU patients, providing valuable insights for a better understanding of CSU pathogenesis.

## 2 MATERIALS AND METHODS

### 2.1 Research objects

A total of 200 patients diagnosed with CSU were selected for the study from The First Affiliated Hospital of Hebei North University between January 2020 and December 2022. Among them, 61 patients met the inclusion criteria, while 17 patients declined to participate, and 2 patients withdrew during the study. Ultimately, 120 patients were included as subjects. Another 120 healthy individuals from the same period were recruited as the control group. Upon enrollment, comprehensive clinical baseline data, including sex, age, and body mass index (BMI) were recorded for all participants. In the CSU group, there were 53 male and 67 female patients, with an average age of 39.48 ± 6.97 years, and a BMI of 24.41 ± 1.33 kg/m^2^. The duration of the disease was 3.37 ± 1.52 years. Pruritus severity, assessed using the visual analogue scale (VAS), was categorized as follows: 58 patients with mild pruritus, 43 patients with moderate pruritus, and 19 patients with severe pruritus. The Urticaria Activity Score (UAS) was used to evaluate disease activity, with 51 patient classified as having mild activity, 41 patients as moderate activity, and 28 patients as severe activity. Based on the Chronic Urticaria Quality of Life Questionnaire (CU‐Q2oL) score of CSU patients, 57 cases were classified as mildly affected, 46 cases were moderately affected, and 17 cases were severely affected. The control group consisted of 120 individuals including 58 males and 62 females with an average age of 38.86 ± 6.25 years and a BMI of 24.30 ± 1.18 kg/m^2^.

### 2.2 Inclusion and exclusion criteria

Inclusion criteria were as follows: patients who met the diagnostic criteria for CSU as outlined in the EAACI/GA(2)LEN/EDF/WAO guidelines for the treatment of urticaria (2013)^17^; age of 18 years or older; no use of antihistamines, glucocorticoids, or immunosuppressants within 1 month; ability to read, communicate effectively and fully participating in this study; absence of an conditions that met the exclusion criteria; and availability of complete and accurate information.

Exclusion criteria were as follows: women who were pregnant or lactating; patients with other types of urticaria; individuals with severe hepatic, renal and other organ insufficiency; those who had coexisting allergic or autoimmune diseases; individuals with severe chronic diseases, systemic lesions or malignancies; individuals who had used antihistamines, glucocorticoids or immunosuppressive drugs within 1 month; individuals with pre‐existing cognitive impairments or communication difficulties that could affect their participation; and individuals with incomplete clinical information.

### 2.3 Blood sample collection

A fasting elbow venous blood of 3 mL was collected from all subjects in the early morning. Blood sample (1 mL) was preserved in an anticoagulant tube containing sodium heparin for the analysis of the Peripheral blood Treg and Th17 cell ratio using flow cytometry. The remaining 2 mL blood was allowed to clot for 30 min at room temperature, followed by centrifugation for 15 min at 1600*g* to separate the serum. The serum was then stored at −80°C for subsequent determination of cytokine levels.

### 2.4 Assessment of skin pruritus intensity

The intensity of pruritus in CSU patients was assessed using the Visual Analog Scale (VAS). A score ranging from 0 to 10 points was assigned based on the severity of pruritus experienced by the patients. A score of 0 indicated the absence of pruritus; while scores of 1–3, 4–6, 7–10 indicated mild, moderate and severe pruritus, respectively.^18^

### 2.5 Disease severity assessment

In accordance with the EAACI/GA (2) LEN/EDF/WAO guidelines for urticaria (2013), UAS7 was utilized to assess CSU severity. The UAS7 is based on a scoring system where the number of wheals is assigned a score from 0 to 3 points, and the intensity of pruritus is also scored from 0 to 3 points. The score is determined for a period of 7 days, with a maximum total score of 42 points; a score of 0–14 points indicates mild CSU, 15–28 points represents moderate CSU, and 29–42 points corresponds to severe CSU.^19^

### 2.6 Assessment of life quality

The assessment of the quality of life of CSU patients was conducted using the CU‐Q2oL questionnaire. The questionnaire consists of 23 items that evaluate various aspects of the patients life, including pruritus, edema, the impact of the disease on daily life, sleep quality, self‐restraint and the influence on appearance. Each item is rated on a 5‐level scale, with scores ranging from 1 to 5 points, corresponding to “not at all,” “somewhat,” “quite a bit,” “a lot,” and “very much,” respectively, to describe the degree of impact. After linear transformation of the raw data, the total score ranged 0–100 points. A higher score indicates a greater impact on the patients life, with 0–33 points indicating mild impact, 34–67 points indicating moderate impact, and 68–100 points indicating severe impact.^20^

### 2.7 Detection of Th17/Treg cell ratio by flow cytometry

Flow cytometry was conducted for analysis of the Th17 and Treg cell numbers. Th17 cell numbers were expressed as CD3+CD4+CD196+ and Treg cell numbers were expressed as CD3+CD4+CD25+FOXP3+.

To performed the flow cytometry analysis, the following procedures were conducted. A total of 1 mL cell suspension (resuspended by phosphate buffered saline [PBS] was treated with 2 μL of Cell Activation Cocktail and incubated for 4 h at 37°C. After incubation, the cells were centrifuged at 350*g* for 5 min and resuspended with 100 μL PBS. For cell labeling, each tube was added with CD3 (5 μL), CD4 (5 μL) and CD25 (5 μL) antibodies and incubated for 30 min at 4°C in the dark. After incubation, the samples were centrifuged for 5 min at 350*g* to remove the supernatant. After PBS washes, the cells were subject to another centrifugation (350*g* for 5 min) to remove the supernatant. To fix the cells, a fixative mixture consisting of True‐Nuclear™ 4X Fix Concentrate and True‐Nuclear™ Fix Diluent was prepared at a ratio of 1:3. The cells were resuspended in 1 mL of the fixative mixture and incubated at room temperature for 50 min, protected from light. After incubation, the cells were centrifuged at 400*g* for 5 min to discard the supernatant. The fixation process was repeated twice. Next, the cells were resuspended with 100 μL Perm Buffer working solution, and 5 μL CD19 and 5 μL FOXP3 antibodies were added to each tube for labeling, followed by incubation for 30 min at 4°C. Subsequently, the cells were centrifuged at 400*g* for 5 min, and the supernatant was removed. The cells were washed twice with 1 mL of PBS, and the supernatant was discarded. Finally, the cells were resuspended with 500 μL PBS, and the levels of CD3+CD4+CD196+ and CD3+CD4+CD25+FOXP3+ were analyzed using a flow cytometer.

The FITC antihuman CD3 antibody (317306), FITC antihuman CD4 antibody (300505), APC antihuman CD196 (CCR6) antibody (353416), APC antihuman CD25 antibody (302609), PE antimouse/rat/human FOXP3 antibody (320007), True‐Nuclear™ Transcription Factor Buffer Se (424401), Permeabilization Wash Buffer (421002), and Cell Actibation Cocktail (423303) were obtained from BioLegend.

### 2.8 Enzyme‐linked immunosorbent assay (ELISA) for serum cytokine levels

Serum levels of interleukin (IL)‐17, IL‐21, IL‐35 and transforming growth factor‐β1 (TGF‐β1) were determined using ELISA. The IL‐17 (PI550), IL‐21 (PI588), and TGF‐β1 (PI880) kits were obtained from Beyotime, while the IL‐35 (E‐EL‐H2443c) kit was purchased from Elabscience. All experimental procedures were performed in strict accordance with the provided kit instructions.

### 2.9 Statistical analysis

Statistical analysis and graphing were conducted using SPSS 21.0 (IBM) and GraphPad Prism 8.01 (GraphPad Software) software. The Shapiro–Wilk test was used to assess the normal distribution of the measured data, and normally distributed data were expressed as mean ± standard deviation. The *t* test was applied for comparisons between two group comparisons. Comparison among multiple groups was performed by one‐way analysis of variance. Post hoc test was conducted using Tukey's multiple comparisons test. Fisher's exact test was employed for analyzing categorical variables. Correlation analysis was performed to evaluate the correlation between the indicators. *p* values were obtained from a two‐sided test. In all statistical references, a value *p* < .05 was considered statistically significant.

## 3 RESULTS

### 3.1 No significant differences in clinical baseline characteristics of all subjects

The clinical baseline characteristics of all subjects were displayed in Table 1. No significant differences were observed in parameters such as age, sex, and BMI between the two groups (*p* > .05), indicating comparability between the groups.

**Table 1:** Comparison of clinical baseline characteristics.

Characteristics	The normal group (*N* = 120)	The CSU group (*N* = 120)	*p* Value
Age (years)	38.86 ± 6.25	39.48 ± 6.97	.442
Sex (cases)			.605
Male	58	53	
Female	62	67	
BMI (kg/m^2^)	24.30 ± 1.18	24.41 ± 1.33	.489

*Note*: Normal distribution data were expressed as mean ± standard deviation and analyzed using the independent samples *t* test. Categorical variables were analyzed using Fisher's exact test. *p* < .05 was considered to demonstrate statistically significant differences.

Abbreviations: BMI, body mass index; CSU, chronic spontaneous urticaria.

### 3.2 Immune dysregulation of Th17/Treg in peripheral blood of CSU patients

**Figure 1:**
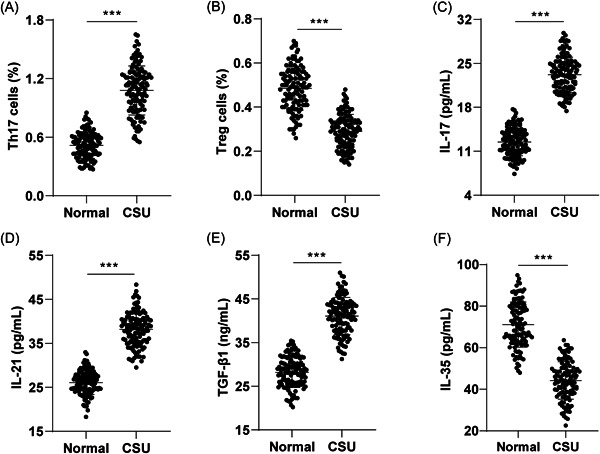
Peripheral blood Th17/Treg immune dysregulation in CSU patients. (A and B) Flow cytometry for the ratio of Th17 and Treg cells in peripheral blood; (C–F) ELISA for serum Th17 cytokine (IL‐17, IL‐21) and Treg cytokine (TGF‐β1, IL‐35) levels. Data were expressed as mean ± standard deviation. Comparison between groups was analyzed using the *t* test. ***p < .001.

Flow cytometry analysis revealed a significant increase in the proportion of Th17 cells and a notable decrease in the percentage of Treg cells in the peripheral blood of CSU patients compared to the normal group (Figure 1A,B, *p* < .001). Additionally, ELISA results indicated significantly elevated levels of IL‐17, IL‐21, and TGF‐β1, while the level of IL‐35 level was notable reduced in the CSU group relative to the normal group (Figure 1C–F, *p* < .001). The preceding results suggest an immune dysregulation in Th17/Treg cells in CSU patients.

### 3.3 Correlation between Th17 and Treg cells in peripheral blood and their cytokines in patients with CSU

**Figure 2:**
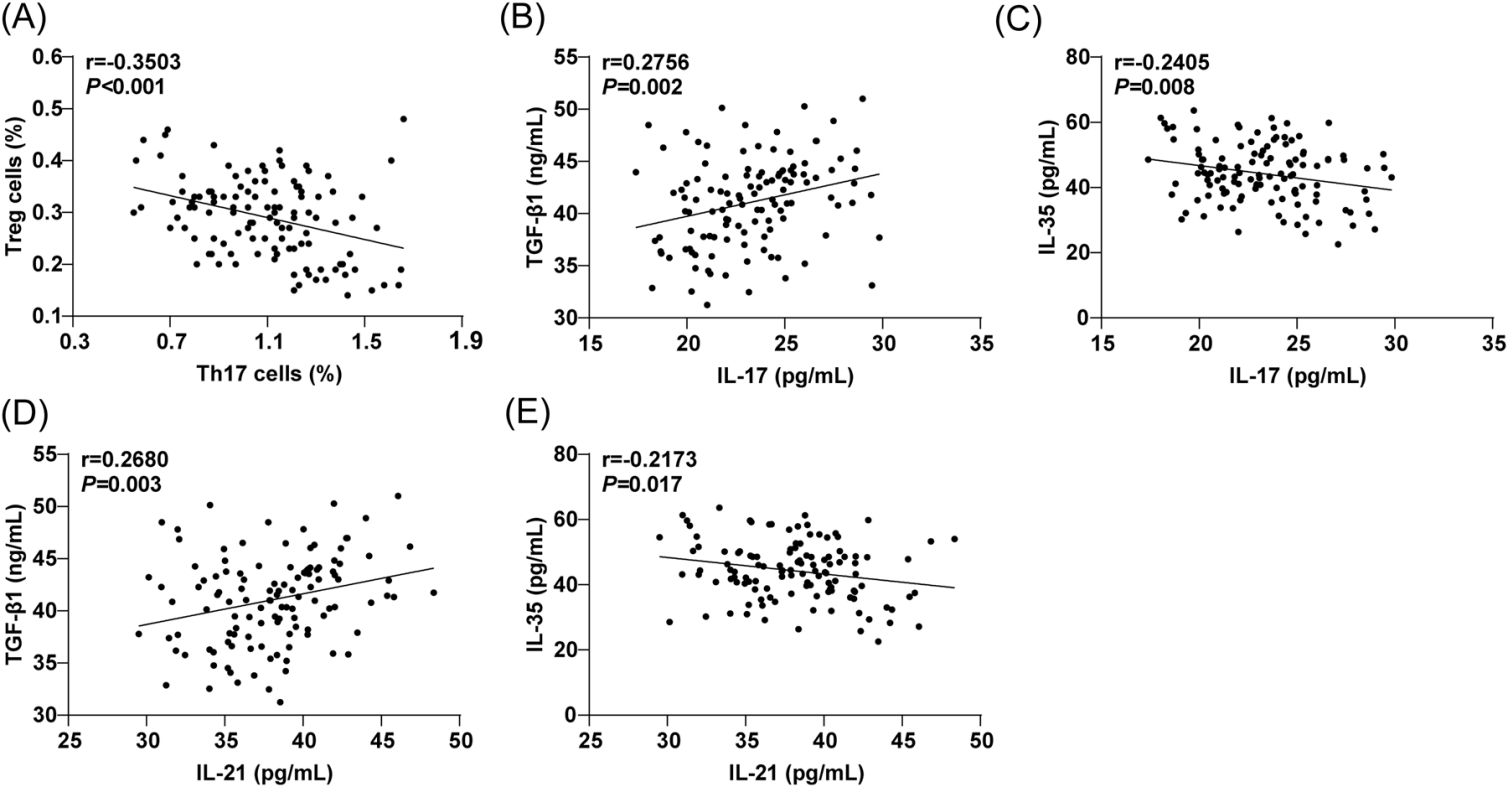
Correlation between Th17 and Treg cells in peripheral blood and their cytokines in patients with CSU. (A) Correlation between peripheral blood Th17 and Treg cell ratios; (B–E) Correlation between Th17 and Treg cytokines in peripheral blood. Pearson's analysis was used to estimate the correlation between peripheral blood Th17 and Treg cells and their related cytokines in CSU patients. *r* represents the correlation coefficients. *p* < .05 indicated a statistically significant difference.

The Pearson's correlation analysis revealed significant inverse correlations between the percentage of Th17 cells in peripheral blood and the percentage of Treg cells in CSU patients (Figure 2A, *r* = −.3503, *p* < .001). Furthermore, the serum TGF‐β1 level showed significant positive associations with IL‐17 (Figure 2B, *r* = .2756, *p* = .002) and IL‐21 levels (Figure 2D, *r* = .2680, *p* = .003). The level of serum IL‐35 exhibited markedly negative correlation with IL‐17 (Figure 2C, *r* = −.2405, *p* = .008) and IL‐21 levels (Figure 2E, *r* = −.2173, *p* = .017), respectively.

### 3.4 Correlation of serum Th17 and Treg cytokines with the pruritus levels in patients with CSU

**Figure 3:**
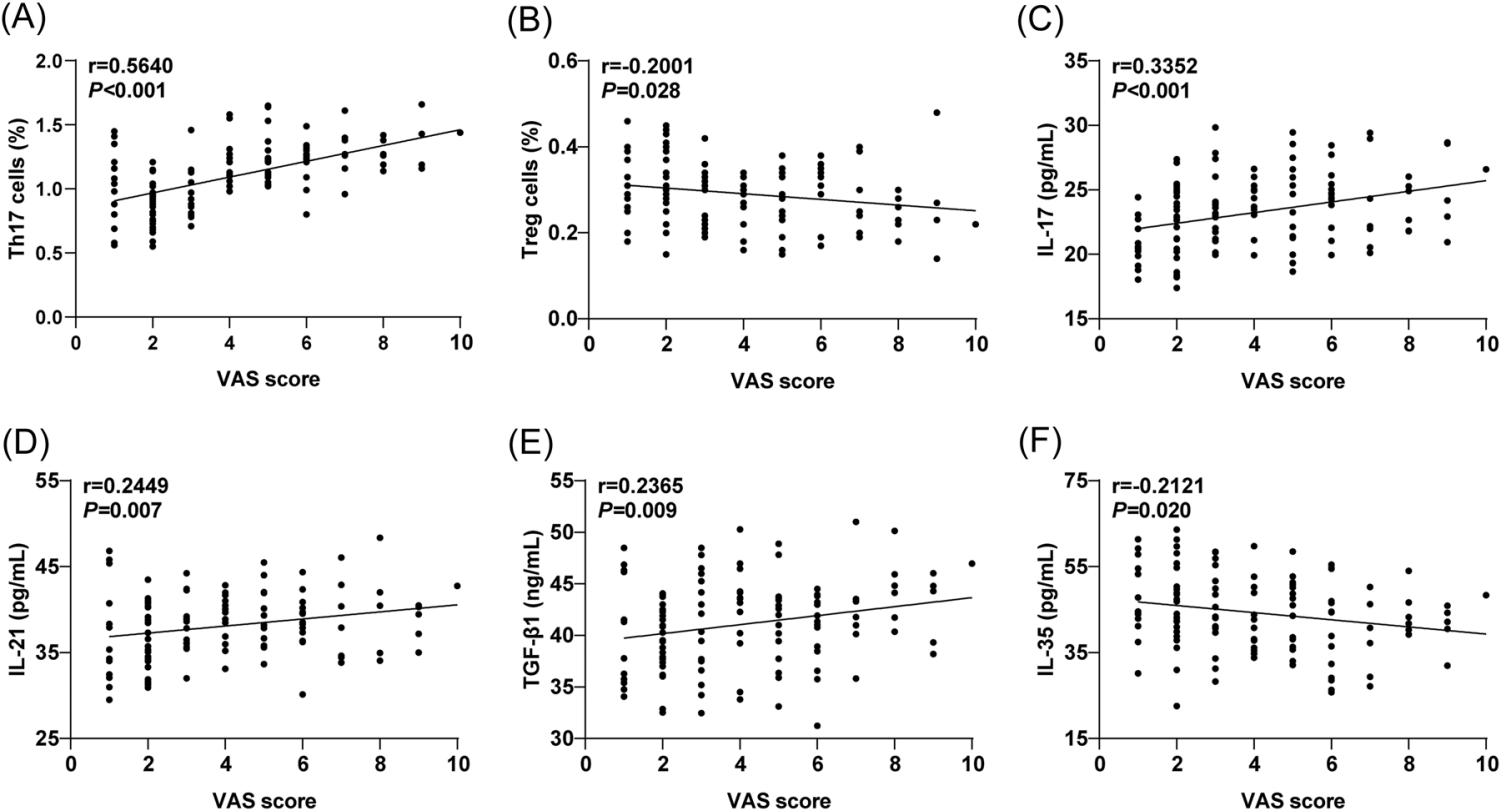
Correlation of serum Th17 and Treg cytokines with the pruritus levels in patients with CSU. The degree of pruritus in CSU patients was assessed by VAS. (A–B) Correlation betwen peripheral blood Th17 and Treg cell ratios and VAS scores; (C–E) Correlation between peripheral blood Th17 and Treg cytokines and VAS scores. Pearson's method was conducted to analyze the correlation between Th17/Treg and the level of pruritus in CSU patients. The correlation coefficient was denoted by *r*, with *p* < 0.05 indicating a statistically significant difference. CSU, chronic spontaneous urticaria.

The CSU patients were categorized into mild pruritus group (*N* = 58), moderate pruritus group (*N* = 43), and severe pruritus group (*N* = 19) based on their VAS scores. As shown in Table 2, the ratio of Th17 cells and the levels of serum IL‐17, IL‐21, and TGF‐β1 were remarkably different among different groups (*p* < .05). Specifically, the percentage of Th17 cells in peripheral blood and the levels of IL‐17, IL‐21, and TGF‐β1 increased with the intensity of pruritus. However, there was no evident difference in the Treg cell percentage in peripheral blood and the level of serum IL‐35 among the three groups (*p* > .05). Pearson's analysis revealed significant positive correlations between the VAS scores of CSU patients and the ratio of Th17 cells in peripheral blood (Figure 3A, *r* = .5640, *p* < .001), as well as the serum levels of IL‐17 (Figure 3C, *r* = .3352, *p* < .001), IL‐21 (Figure 3D, *r* = .2449, *p* = .007) and TGF‐β1 (Figure 3E, *r* = .2365, *p* = .009). Additionally, there were significant negative correlations between the VAS score and the proportion of Treg cell in peripheral blood (Figure 3B, *r* = −.2001, *p* = .028), as well as the level of serum IL‐35 (Figure 3F, *r* = −.2121, *p* = .020).

**Table 2:** Expression of serum Th17/Treg cytokines in CSU patients with different pruritus levels.

Indicators	The CSU group (*N* = 120)			*p* Value
	Mild pruritus (*N* = 58)	Moderate pruritus (*N* = 43)	Severe pruritus (*N* = 19)	
Th17 (%)	0.94 ± 0.22	1.22 ± 0.19	1.30 ± 0.17	**<.001**
Treg (%)	0.31 ± 0.08	0.28 ± 0.07	0.27 ± 0.08	.087
IL‐17 (pg/mL)	22.41 ± 2.75	23.95 ± 2.61	24.31 ± 3.03	**.005**
IL‐21 (pg/mL)	37.09 ± 4.08	39.00 ± 3.09	39.33 ± 4.21	**.016**
TGF‐β1 (ng/mL)	40.13 ± 4.19	41.37 ± 4.19	43.33 ± 3.85	**.014**
IL‐35 (pg/mL)	46.30 ± 9.43	42.54 ± 8.90	41.89 ± 6.88	.054

*Note*: Data were expressed as mean ± standard deviation and analyzed using one‐way analysis of variance with Tukey's multiple comparisons test as post hoc analysis. Statistical significance was defined as *p* < .05 are in bold.

Abbreviation: CSU, chronic spontaneous urticaria.

### 3.5 Correlation of serum Th17/Treg cytokines with the disease severity in CSU patients

**Figure 4:**
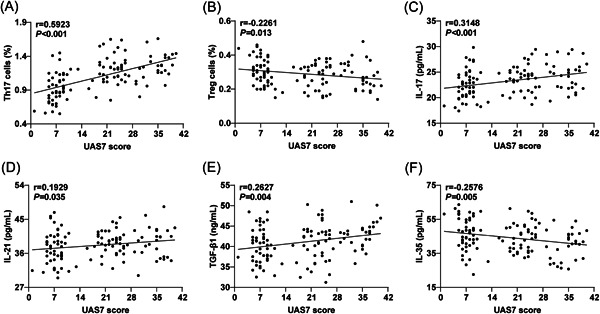
Correlation of serum Th17/Treg cytokines with the disease severity in CSU patients. The degree of disease in CSU patients was estimated by UAS7. (A–B) Correlation between peripheral blood Th1/Treg cell ratio and UAS7 score; (C–F) Correlation between peripheral blood Th17/Treg cytokines and UAS7 score. Pearson's method was carried to analyze the correlation between serum Th17/Treg cytokines and the degree of disease in CSU patients. *r* was the correlation coefficient. *p* < .05 indicated that the difference was statistically significant. CSU, chronic spontaneous urticaria.

CSU patients were classified into mild (*N* = 51), moderate (*N* = 41), and severe (*N* = 28) groups, based on their UAS7 score. According to the results in Table 3, there were significant differences in the proportion of Th17 cells in peripheral blood and the levels of serum IL‐17, TGF‐β1, and IL‐35 among the three groups (all *p* < .05): Th17 cell proportion and the expression of IL‐17 and TGF‐β1 expression increased with disease progression, while the expression of IL‐35 decreased. However, there was no evident difference in the ratio of Treg cells in peripheral blood and the level of serum IL‐21 among the three groups (all *p* > .05). Subsequently, with regard to the results of Pearson's correlation analysis revealed significant positive correlations between the UAS7 scores of CSU patients and the proportion of Th17 cells in peripheral blood (Figure 4A, *r* = .5923, *p* < .001), as well as the serum levels of IL‐17 (Figure 4C, *r* = .3148, *p* < .001), IL‐21 (Figure 4D, *r* = .1929, *p* = .035), and TGF‐β1 (Figure 4E, *r* = −.2627, *p* = .004). However, the ratio of Treg cells in peripheral blood (Figure 4B, *r* = −.2261, *p* = .013), and the level of serum IL‐35 level (Figure 4F, *r* = −.2576, *p* = .005) were notably negatively correlated with the UAS7 scores of CSU patients.

**Table 3:** Expression of serum Th17/Treg cytokines in CSU patients with different disease severity.

Indicators	The CSU group (*N* = 120)			*p* Value
	Mild (*N* = 51)	Moderate (*N* = 41)	Severe (*N* = 28)	
Th17 (%)	0.93 ± 0.21	1.21 ± 0.21	1.24 ± 0.19	**<.001**
Treg (%)	0.31 ± 0.08	0.28 ± 0.07	0.28 ± 0.08	.159
IL‐17 (pg/mL)	22.32 ± 2.83	23.62 ± 2.50	24.47 ± 2.88	**.003**
IL‐21 (pg/mL)	37.19 ± 4.24	38.81 ± 2.99	38.84 ± 4.09	.073
TGF‐β1 (ng/mL)	40.14 ± 4.10	40.87 ± 4.64	43.11 ± 3.30	**.010**
IL‐35 (pg/mL)	46.38 ± 9.49	44.74 ± 7.75	39.68 ± 8.58	**.006**

*Note*: Data were expressed as mean ± standard deviation. Data were analyzed using one‐way analysis of variance, followed by post hoc analysis using Tukey's multiple comparisons test. Differences were considered to be significant at *p* < .05 are in bold.

Abbreviation: CSU, chronic spontaneous urticaria.

### 3.6 Correlation of serum Th17/Treg cytokines with the life quality in CSU patients

**Figure 5:**
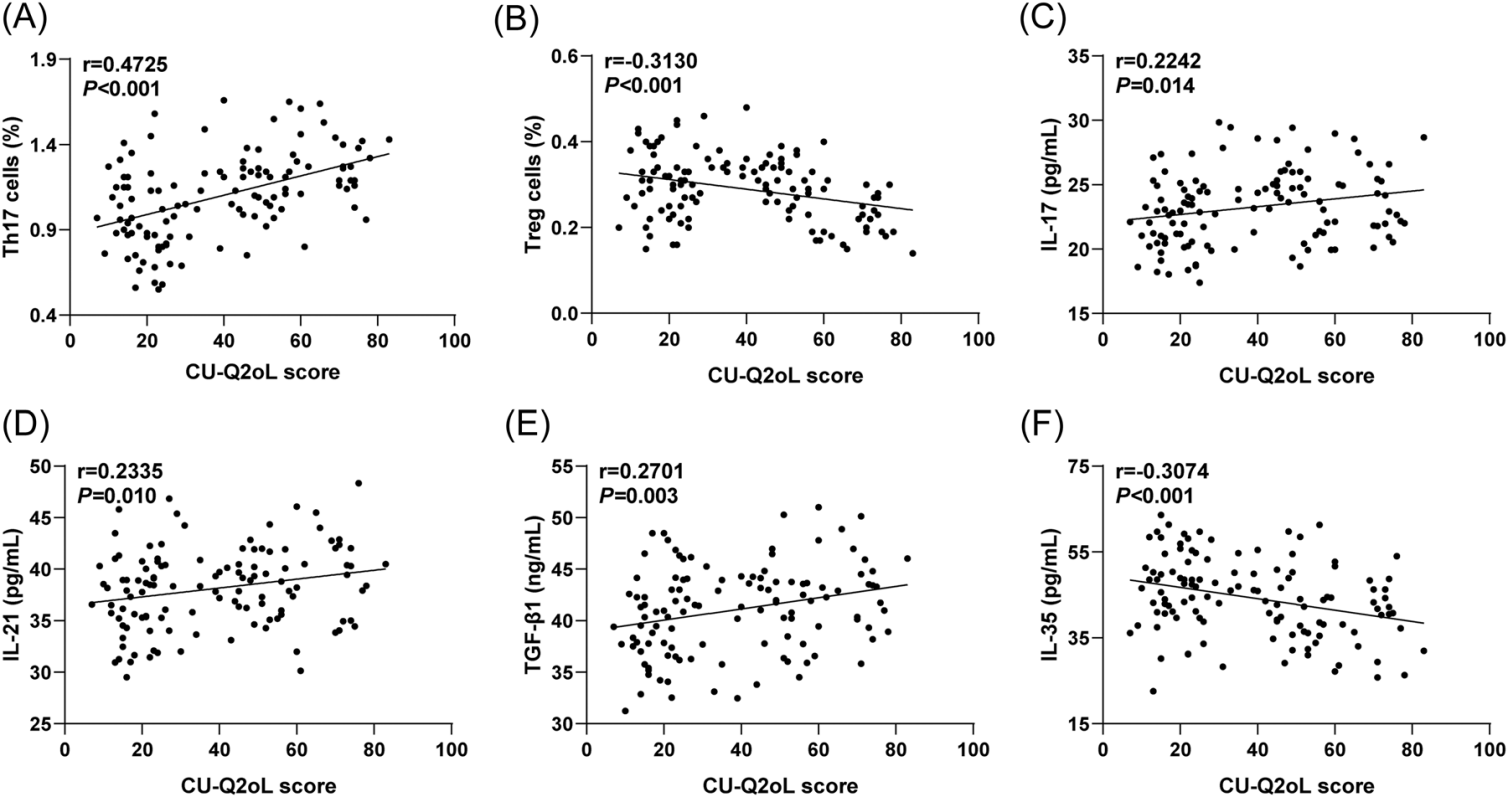
Correlation of serum Th17/Treg cytokines with quality of life in CSU patients. CU‐Q2oL was employed to assess the quality of life of CSU patients. (A–B) Correlation between peripheral blood Th17/Treg cell ratio and CU‐Q2oL score; (C–F) Correlation between Th17/Treg cytokines in peripheral blood and CU‐Q2oL score. The correlation between serum Th17/Treg cytokines and the quality of life of CSU patients was analyzed by Pearson. *r* was used as the correlation coefficients. *p* < .05 was considered to demonstrate statistically significant differences.

CSU patients were categorized into three groups based on their CU‐Q2oL scores: mildly affected (*N* = 57), moderately affected (*N* = 46), and severely affected (*N* = 17) groups. As depicted in Table 4, there were significant differences in the ratio of Th17 and Treg cells and the levels of serum IL‐17, TGF‐β1, and IL‐35 among the three groups (*p* < .05). Specifically, as the quality of life decreased, the proportion of Th17 cells and IL‐17 and TGF‐β1 levels increased, while the proportion of Treg cells and the level of IL‐35 decreased. However, no distinct difference was identified in the level of serum IL‐21 among the three groups (*p* > .05). Pearson method confirmed significant relationships between the CU‐Q2oL score of CSU patients and the proportion of Th17 cells in peripheral blood (Figure 5A, *r* = .4725, *p* < .001), the levels of serum IL‐17 (Figure 5C, *r* = .2242, *p* = .014), IL‐21 (Figure 5D, *r* = .2335, *p* = .010), and TGF‐β1 (Figure 5E, *r* = .2701, *p* = .003) positively. Conversely, the CU‐Q2oL score of CSU patients was significantly negatively associated with the percentage of Treg cells in peripheral blood (Figure 5B, *r* = −0.3130, *p* < .001) and the level of serum IL‐35 (Figure 5F, *r* = −.3074, *p* < .001).

**Table 4:** Expression of serum Th17/Treg cytokines in CSU patients with different quality of life.

Indicators	The CSU group (*N* = 120)			*p* Value
	Mildly affected (*N* = 57)	Moderately affected (*N* = 46)	Severely affected (*N* = 17)	
Th17 (%)	0.96 ± 0.23	1.21 ± 0.22	1.25 ± 0.14	**<.001**
Treg (%)	0.30 ± 0.08	0.30 ± 0.07	0.23 ± 0.05	**.001**
IL‐17 (pg/mL)	22.55 ± 2.89	24.09 ± 2.76	23.41 ± 2.42	**.022**
IL‐21 (pg/mL)	37.35 ± 4.07	38.63 ± 3.43	39.39 ± 4.00	.086
TGF‐β1 (ng/mL)	40.09 ± 4.33	41.75 ± 4.16	42.59 ± 3.65	**.040**
IL‐35 (pg/mL)	46.66 ± 9.04	42.68 ± 8.71	40.44 ± 8.03	**.013**

*Note*: Data were expressed as mean ± standard deviation and analyzed using one‐way analysis of variance. Tukey's multiple comparisons test was used for post hoc analysis. *p* < .05 indicated a statistically significant difference are in bold.

Abbreviation: CSU, chronic spontaneous urticaria.

## 4 DISCUSSION

CSU is a debilitating skin disease that significantly impacts the daily life of affected individuals.^21^ It affects approximately 1% of the global population, CSU has a substantial economic burden due to work productively impairment, with more than 20% of those with CSU patients experiencing frequent work absence a productivity reduction of 27%.^22^ Consequently, there is an urgent need to understand CSU pathogenesis to develop improved diagnostic and treatment strategies for patients. However, the relationship between Th17/Treg cells imbalance and CSU pathogenesis remains poorly investigated. In light of this literature, our study focused on the correlation between Th17/Treg immune dysregulation and CSU. Our findings demonstrated a significant association between the proportion of Th17 cells and the levels if IL‐17, IL‐21, and TGF‐β1 in CSU patients with pruritus intensity, disease severity, and quality of life, while the proportion of peripheral blood Treg cells and serum IL‐35 level exhibited a negative correlation with pruritus intensity, disease severity, and quality of life.

Th17/Treg immune dysregulation has emerged as a crucial factor in the development of several skin diseases, including hidradenitis suppurativa^23^ and atopic dermatitis.^24^ Our experimental results prompted us to investigate the potential involvement of Th17/Treg in the pathogenesis of CSU. One study conducted by Cho et al.^25^ revealed that Th17 cell‐mediated immune responses facilitate mast cell proliferation through the action of keratinocyte‐derived stem cell factor. Additionally, Th17 cell subsets have been implicated in promoting fibrosis, autoimmunity, and inflammation in systemic sclerosis, while Treg cell subsets exert immunosuppressive effects and regulate the activity of Th17 cells.^26^ Furthermore, Treg cells play a vital role in counter‐regulating excessive immune responses and preventing the development of autoimmune diseases.^27^ An existing study has demonstrated the activation of Treg cell subpopulations, specifically Foxp3^+^ T cells, can effectively suppress the activity of Th1 and Th17 cells.^28^ Notably, dysregulation of Th17 cells and Treg cells with an increase in Th17 cell populations and a decrease in Treg cell populations has been observed in systemic lupus erythematosus.^29^ Our study aligns with these observations, as we identified an elevated proportion of Th17 cells and a reduced proportion of Treg cells in CSU patients.

IL‐22 and IL‐17 are crucial cytokines involved in the immune defense of psoriatic plaques through their ability to facilitate the production of antimicrobial proteins.^30^ Additionally, IL‐21, a cytokine primarily produced by Th17 cells, has been implicated in the development of autoimmune diseases.^31^ IL‐35, predominantly derived from regulatory cells, has emerged as a promising therapeutic approach for pemphigus.^32^ Besides, a shift in the Th17/Treg cell balance towards the Treg cell side has been shown to lead to increased levels of IL‐10 and TGF‐β, while decreasing IL‐17 levels.^11^ The treatment of gallic acid modulates the Th17/Treg cell balance, resulting in increased expression of IL‐10 and TGF‐β, and reduced levels of IL‐17.^33^ Consistent with previous studies, our findings demonstrated significant positive correlations between serum TGF‐β1 and IL‐17 and IL‐21 levels in CSU patients, while serum IL‐35 levels exhibited significant negative correlations with IL‐17 and IL‐21 levels.

According to the study conducted by Atwa et al.^34^ it was observed that CSU patients exhibited significantly elevated levels of Th17 cytokines compared to healthy individuals. Notably, patients with severe disease demonstrated higher levels of IL‐17, indicating its potential role in disease severity.^35^ Sekumab, an IL‐17 neutralizing agent, has shown efficacy in treating moderate‐to‐severe spinalarthritis and psoriasis, and has also demonstrated positive outcomes in refractory CSU patients who did not respond to omalizuma.^36^ Furthermore, Treg cells, which play a regulatory role in immune responses, have been implicated in the pathogenesis of CSU. Studies have reported reduced expression of Treg cells in CSU patients.^37^ Consistent with these findings, our study revealed a significant positive correlation between peripheral blood Th17 cells and pruritus level, disease severity, and impaired quality of life in CSU patients. Conversely, Treg cells exhibited a significant negative correlation pruritus level, disease severity, and quality of life in CSU patients. These results suggest that the dysregulation of Th17/Treg in peripheral blood may contribute to the pathogenesis of CSU.^38^ Similar associations between Th17/Treg cells and disease severity has been reported in other skin diseases, such as atopic dermatitis, where the ratio of Th17 cells is positively associated with disease severity, while the proportion of Treg cells is inversely correlated.^39^ In psoriasis, the absence of Treg cells has been shown to contribute the disease maintenance and exacerbation.^40^ Collectively, these findings indicate the potential involvement of Th17/Treg immune dysregulation in the pathogenesis of CSU.

In our study, we investigated the relationship between serum Th17/Treg immune dysregulation and the severity of CSU. Our findings revealed a significant positive correlation between the percentage of Th17 cells in peripheral blood, as well as the levels of serum IL‐17, IL‐21, and TGF‐β1, with the degree of pruritus, disease severity, and impaired quality of life in CSU patients. Conversely, the percentage of Th17 cells in peripheral blood and the level of serum IL‐35 showed a notable negative correlation pruritus level, disease severity, and quality of life. These results suggest that Th17/Treg immune dysregulation may play a crucial role in the pathogenesis of CSU. However, it is important to acknowledge the limitations of our study. First, the sample small was relatively small, which may limit the generalizability of our findings. Future studies with larger sample sizes are warranted to validate our results. Second, our study focused solely on the correlation between serum Th17/Treg immune dysregulation and disease severity in CSU patients, the underlying mechanism of CSU onset and progression remains unclear. In future research will focus on expanding the sample size, conducting multicenter studies, and investigating the mechanism underlying CSU pathogenesis to provide novel therapeutic approaches for the treatment of CSU patients in clinical practise.

## AUTHOR CONTRIBUTIONS

Xiaojing Yang contributed to the study concepts, study design. Leigang Chen contributed to the literature research. Xiaojing Yang, Leigang Chen, Shining Wang, and Yuanhui Wu contributed to the experimental studies and data acquisition. Xiaojing Yang, Xiangzhao Zhou, and Zhaoying Meng contributed to the data analysis and statistical analysis; Xiaojing Yang contributed to the manuscript preparation and Leigang Chen contributed to the manuscript editing and review. All authors read and approved the final manuscript.

## ETHICS STATEMENT

The current study was authorized by the academic ethics committee of The First Affiliated Hospital of Hebei North University (Approval no. K2019167). All experimental procedures were performed in strict accordance with Declaration of Helsinki. All subjects were informed fully of the study objective and signed the informed consent before sampling.

## Data Availability

All data generated or analyzed during this study are included in this article. Further enquiries can be directed to the corresponding author.
